# Partner Effects of Childhood Maltreatment: A Systematic Review and Meta-Analysis

**DOI:** 10.1177/15248380231173427

**Published:** 2023-05-20

**Authors:** Marie-Pier Vaillancourt-Morel, Ève-Line Bussières, Marie-Chloé Nolin, Marie-Ève Daspe

**Affiliations:** 1 Université du Québec à Trois-Rivières, QC, Canada; 2 Université de Montréal, QC, Canada

**Keywords:** childhood maltreatment, couple, partner effect, abuse, neglect, dyadic, review, meta-analysis

## Abstract

Although several studies have shown that childhood maltreatment (CM) is associated with a host of negative consequences including romantic relationship difficulties for victims in adulthood, most overlooked the potential effects on the romantic partner. This systematic review and meta-analysis aims to comprehensively synthesize the literature on the association between a person’s CM and their partner’s individual and couple outcomes. We searched PubMed, PsycNET, Medline, CINAHL, and Eric using search strings related to CM and partner. We identified 3,238 articles after removal of duplicates; 28 studies met the inclusion criteria and relied on independent sample. The studies reported associations between a person’s CM and a wide breadth of partner’s negative couple outcomes (e.g., communication, sexuality) as well as intra-individual psychological difficulties (e.g., psychological distress, emotion, and stress reactivity). Meta-analytic results showed significant, but trivial to small associations between a person’s CM and their partner’s lower relationship satisfaction (*r* = −.09, 95% CI [−.14, −.04]), higher intimate partner violence (*r* = .08, [.05, .12]), and higher psychological distress (*r* = .11, [.06, .16]). These associations were similar for women and men and did not differ as a function of sample’s mean age, proportion of cultural diversity, and publication year. These findings suggest that a person’s CM is related to their partner’s outcomes including to the partner’s intra-individual outcomes. Prevention and intervention strategies should acknowledge that a person’s CM may also affect their romantic partner, considering the couple as a reciprocal system, and offer victims’ romantic partners specific services.

Childhood maltreatment (CM) refers to all forms of abuse and neglect of children, including childhood sexual abuse (CSA), physical abuse (CPA), emotional abuse (CEA), physical neglect (CPN), and emotional neglect (CEN) (World Health Organization, 2019). In large population-based studies from North America, 35% to 40% of individuals retrospectively report at least one form of CM (Cyr et al., 2013; MacDonald et al., 2016), with a series of meta-analysis documenting high prevalence rates worldwide (12.7% CSA, 16.3% CPN, 18.4% CEN, 22.6% CPA, 36.3% CEA; Stoltenborgh et al., 2015). Specific forms of CM rarely occur in isolation as multiple chronic victimizations is frequent (Cyr et al., 2013; MacDonald et al., 2016). Numerous studies, including important reviews and meta-analyses, have shown that CM is associated with a host of negative consequences for victims in adulthood, including psychopathology, posttraumatic stress, substance use disorders, and poor health outcomes (Chandan et al., 2020; Edalati & Krank, 2016; Lewis et al., 2021; Schar et al., 2022).

CM encompasses relational traumas, whereby the betrayal, powerlessness, or disregard experienced have the potential to disturb future romantic relationships (Briere, 2002; Colman & Widom, 2004). Accumulating evidence suggests that all forms of CM are associated with victims’ difficulties in several aspects of romantic relationships including intimacy disturbance (DiLillo et al., 2009; Vaillancourt-Morel et al., 2019), intimate partner violence (IPV; Godbout et al., 2019), sexual difficulties and dissatisfaction (Vaillancourt-Morel et al., 2021), relationship dissatisfaction, and dissolution (Colman & Widom, 2004; Vaillancourt-Morel et al., 2015, 2021). A recent review of 43 articles using cross-sectional and longitudinal designs indicated that all types of CM are linked to victims’ lower relationship quality in men and women in community, college, and clinical samples (Zamir, 2021). Recent meta-analyses also showed that emotional maltreatment is negatively related to victims’ romantic relationship well-being (*r* = .14; Cao et al., 2020) and that CM is related to higher IPV victimization (*r* = .18; Li et al., 2019).

Although several studies as well as developmental and trauma theories contend how CM may affect romantic relationships (Bowlby, 1969; Briere, 2002; Finkelhor & Browne, 1985), they mostly focus on the effects of CM on the primary victim—the person who directly experienced CM, and mostly overlooked how CM may affect those closest to the victim, for example, the romantic partner. Yet, outside of the literature on CM, several terms have been used to describe how a person’s traumatic stress may affect the romantic partner, children, and professional helpers of trauma victims including compassion fatigue (Figley, 2002), vicarious victims (Jehu, 1988), and trauma transmission (Baranowsky et al., 1998). In addition, secondary traumatic stress involves feelings of “tension and distress directly related to the demands of living with and caring for someone who displays the symptoms of posttraumatic stress disorder” (Figley, 1998, p. 7).

The Couple Adaptation to Traumatic Stress (CATS) model (Nelson & Smith, 2005; Nelson & Wampler, 2000) outlines that traumatic experiences may be related to (a) symptoms in the primary trauma victim, (b) secondary trauma symptoms in the partner, and (c) dysfunctional relational dynamics within the couple system. Thus, this model first suggests that partners may report individual levels of functioning that are similar to the primary victim’s trauma response including emotional, behavioral, cognitive, and biological symptoms (Nelson & Smith, 2005). Multiple clinical case studies suggest that CM is associated with partners’ negative outcomes via feelings that parallel the victim’s mixed emotional responses and cognitive biases including higher psychological distress and stress reactivity, inadequate affect regulation and mentalization skills, and biased internal representations of self and others (Barnett, 1993; Briere, 2002; MacIntosh, 2013, 2019). This model also suggests that, as the CM occurs within a relational context, the consequences often contaminate other interpersonal intimate relationships with couples that have experienced CM reporting dynamics that are related to the trauma including role disruption, poorer family adjustment, difficulties with intimacy, lower relationship cohesion and satisfaction, greater conflict, anger, and violence (Nelson & Smith, 2005). Cycles of repetition and enactment of past trauma in the couple dynamics that often lead to couple instability and conflicts have been reported in several clinical case studies (MacIntosh, 2017). Thus, based on theoretical and clinical observations, the victim, their partner, and the couple dynamic may all be affected by CM (Nelson & Smith, 2005). However, whether the partners of CM victims report secondary intra-individual trauma symptoms, whether it is their romantic functioning that is affected, or both, remains unclear.

To examine how a person’s CM is related to their partner’s individual and couple outcomes, we need more studies that collect dyadic data, that is, sampling both partners instead of only individuals, and analyzed the association between one’s CM and their partner’s outcome (i.e., partner effect). Recent reviews still report that dyadic studies are scare even if they have significantly increased in the past years (Bigras et al., 2021; Zamir, 2021). Some dyadic studies show that partners of CM victims report lower relationship satisfaction, lower sexual satisfaction, higher IPV victimization and perpetration, and higher individual stress symptoms (DiLillo et al., 2016; Maneta et al., 2012; Nelson & Wampler, 2000; Vaillancourt-Morel et al., 2021; Whisman, 2014). Conversely, other studies have failed to find significant associations between one person’s CM and their partner’s relationship satisfaction and trauma symptoms (Evans et al., 2014; Fritz et al., 2012; Georgia et al., 2018; Nguyen et al., 2017). These mixed findings leave little knowledge on the diversity of partner’s outcomes that may be affected by a person’s CM and the strength of the partner effects. If the partner effects are significant only for romantic functioning outcomes (e.g., relationship satisfaction, IPV), it would suggest that it is the relational dynamic within the couple system that is affected. If the partner effects are significant only for partners’ intra-individual outcomes (e.g., psychological distress), it would support secondary trauma processes. However, it remains unclear whether these partner effects are significant only for romantic functioning outcomes, only for partners’ intra-individual outcomes, or both.

Although a few literature reviews focusing on specific outcomes (e.g., sexuality, relationship satisfaction) included dyadic studies (Bigras et al., 2021; Pulverman et al., 2018; Zamir, 2021), none focused specifically on the partner effect of a person’s CM. For instance, in a systematic review on CM and relationship quality including 43 empirical studies, 9 studies employed dyadic data analysis that allow the examination of partner effect, but the results of these partner associations were not systematically reported (Zamir, 2021). To our knowledge, only one meta-analysis included the effect size of the partner effect (Cao et al., 2020). This meta-analysis, which included 23 studies examining the association between childhood emotional maltreatment (CEA and CEN) and romantic relationship well-being, reported that seven studies used dyadic data and four reported separately the partner effect with an overall significant but small effect size (*r* = −.13, 95% CI [−.21, −.05]; Cao et al., 2020). However, this effect size, based on four studies, included only childhood emotional maltreatment and partner’s outcomes related to the couple’s well-being. A more comprehensive examination of all studies using dyadic data to examine the associations between a person’s CM and their partner’s individual and couple outcomes is important to understand the needs of CM victims’ partners and develop appropriate preventive and intervention strategies that also target the partner and the couple as a system.

## Current Study

The main goal of this systematic review and meta-analysis is to synthesize the existing literature on the associations between a person’s CM and their partner’s outcomes. The first aim is the systematic review. For this aim, we systematically reviewed the existing literature on the associations between a person’s CM and their partner’s outcomes including all potential outcomes (e.g., couple functioning, cortisol response, posttraumatic stress, emotion regulation strategies). This aim offers an overall overview of the diversity of partner’s outcomes that may be affected by a person’s CM. Moreover, it synthetizes these potential effects by organizing the available results based on partner’s outcomes (i.e., whether it represents a romantic functioning outcome or an intra-individual outcome) to qualitatively examine whether these partner effects are significant only for couple outcomes or also for partners’ intra-individual outcomes. The second aim is the meta-analyses. For this aim, based on the results of the systematic review, meta-analyses were conducted for specific outcomes for which at least four studies provided appropriate data on the same, or very similar, concept. This aim allows to quantitatively synthetize the strength of partner effects for specific outcomes that share common features and for which sufficient effect sizes are available. Three specific outcomes provided sufficient effect sizes to conduct meta-analyses. Thus, for this second aim, we estimated the average effect size for the associations between a person’s CM and their partner’s (a) relationship satisfaction, (b) IPV, and (c) psychological distress. As some past studies reported that the effects of CM on victims were different between women and men (Gershon et al., 2008), we also examined, in the meta-analyses, whether gender moderated the examined associations. Moreover, as some differences in the study design may contribute to the ability to detect significant partner effects and explain past mixed findings, we also examined, in the meta-analyses, whether methodological characteristics moderated the examined associations.

## Method

### Protocol and Registration

This protocol was not registered and was conducted according to the Preferred Reporting Items for Systematic Reviews and Meta-Analyses (PRISMA; Page et al., 2021) statement.

### Eligibility Criteria

To be eligible for the systematic review and the meta-analyses, studies had to: (a) report the association between any form of CM and any outcomes in the victim’s partner, (b) assess any form of CM before 18 years of age, (c) examine the effects of CM as compared to the absence of CM (i.e., include participants with and without CM histories), (d) include a sample or a subsample consisting of couples, which is indispensable to examine partner effects, (e) be written in English or French, and (f) be published in a peer-reviewed journal. Moreover, for the meta-analysis only, selected articles had to (g) report sufficient data to allow computation of effect sizes for the partner effects. Participants could be of any age and no restrictions on year of publication were applied. Studies on trauma, adverse childhood experiences, and harsh parenting were included if any type of CM was explicitly assessed. We excluded gray literature (i.e., all materials and research that have not been published through traditional means, including reports and theses), studies which exclusively used a qualitative method of research, as well as case studies. For the meta-analysis, to avoid duplication of information, if several articles reported results from the same sample or if a study presents more than one effect size included in the same meta-analysis (e.g., for different types of CM or different outcomes) all effect sizes from the same study were collapsed into one effect size (i.e., average effect size) (Lipsey & Wilson, 2001). Thus, each participant was included in the same meta-analysis only once.

### Literature Search

The selection of relevant published peer-reviewed articles was based on a variety of strategies. The electronic literature search was conducted using PubMed, PsycNET (PsycINFO, PsycARTICLES), Medline, CINAHL, and Eric for peer-reviewed journal articles. The search strings consisted of multiple combinations of two main components: (a) CM (i.e., child* maltreatment, child* trauma, child* abuse, neglect, child* sexual abuse, child* physical abuse, child* emotional abuse, and child* psychological abuse) and (b) partner (i.e., couple, dyadic, partner, romantic relationship, intimate relationship, actor-partner interdependence model, dyadic analysis). Then, duplicates were removed in a reference management program (EndNote) to facilitate the screening process. All titles, abstracts, and full texts were independently screened by two research assistants and disagreements were discussed with the first author until consensus. Then, the reference lists of selected articles (*k* = 34), relevant systematic and narrative reviews (*k* = 8), the first four pages of results on Google Scholar, and the Google Scholar profile of researchers in this research area (*n* = 9) were thoroughly examined for additional relevant articles that had not been found in the main search. These searches were conducted from August to October 2021 and updated in March 2023.

### Data Collection Process

A structured coding scheme was developed into a Microsoft Excel spreadsheet to ensure consistent extraction of data. This codebook included information related to: (a) publication (i.e., authors, year), (b) sample (i.e., total sample size, age in years, relationship length, proportion of women, proportion of participants from sexual and cultural diversity), (c) methodology (i.e., sample method, research design, type of CM assessed, type of outcomes), and (d) main results (description of main findings, effect sizes if associations with relationship satisfaction, IPV, or psychological distress was assessed). An outcome was classified as a romantic functioning outcome if it refers to couples’ dynamic, interaction, or functioning or if it mostly involved romantic partners (e.g., romantic attachment, communication styles or sexuality with romantic partners). An outcome was classified as an intra-individual outcome if it considered the individual only (e.g., anxiety, depression, emotion dysregulation). After this general classification, we organized the available outcomes in each category based on the similarities between them to facilitate the narrative presentation of results. Two members of the research team (M.-P.V.-M. and M.-C.N.) independently extracted data from each identified article and disagreements were discussed until consensus. For the meta-analysis, data (e.g., correlations) not included in the identified articles was obtained from the corresponding author.

### Data Synthesis

To give an overview of the diversity of partner’s outcomes, all potential outcomes (except the three included in the meta-analysis) were included in the systematic review and synthetized in narrative form. Indeed, given the heterogeneity of outcomes, it was impossible to quantitatively combine these outcomes. Given the diversity of outcomes included in the systematic review, the results are presented based on similarity between the outcomes included and organized based on whether it represents a romantic functioning outcome or an intra-individual outcome. Within these categories, outcomes that share similarity (e.g., related to emotion regulation, communication styles) were presented together. Then, based on the results of the systematic review, meta-analyses were conducted for specific outcomes for which at least four studies provided appropriate data on the same, or very similar, concept. Thus, if a study reported an association between CM and relationship satisfaction, IPV, or psychological distress, then the relevant effect size was included in the related meta-analysis. Studies that described their outcome measure as relation satisfaction or couple satisfaction were included in the relationship satisfaction meta-analysis. Studies that described their outcome measure as IPV, dating violence, dating abuse regardless of whether it was perpetration or victimization were included in the IPV meta-analysis. Studies that described their outcome measure as depression, anxiety, stress or distress, trauma symptoms, posttraumatic stress symptoms or disorder were included in the psychological distress meta-analysis. A single effect size was calculated and allowed per meta-analysis, ensuring that estimates were independent.

### Effect Size Calculations

The Comprehensive Meta-Analysis Software Version 3.0 (Borenstein et al., 2013) was used to perform all the analyses related to the meta-analysis. We decided to use the *r*-Pearson correlation as effect size as it was the most commonly reported effect size among the selected studies and given it is the most informative in terms of the magnitude of associations between CM and partner outcomes. Other available statistics (e.g., means in each group, risk ratios, beta coefficients) were transformed into a Pearson *r* coefficient (for more details, see Borenstein et al., 2021). Effect size magnitude was estimated based on Cohen’s (1988) guidelines, where *r* > .10 is considered small, *r* > .30 medium, and *r* > .50 large. Multiple effect sizes were calculated when studies provided results for different associations (e.g., different forms of CM, different outcomes, men and women separately). When a study reported multiple associations for the same outcome (e.g., EN and relationship satisfaction and EA and relationship satisfaction), a mean effect size was computed for each study by averaging effect sizes for the same outcome within the study (Lipsey & Wilson, 2001). Because of the variability within methods, settings, recruitment procedures, and sample types, all effect size results are reported for a random effects approach which assumes that the studies were not necessarily conducted in the same way (Borenstein et al., 2010; Card, 2012). Heterogeneity was formally assessed with the Q and I^2^ statistics. To determine if the variability in the global effect size could be explained by specific moderators, homogeneity analyses across subgroups were conducted. Specifically, we investigated whether gender, sample’s mean age, proportion of cultural diversity, and year of publication moderated the overall relationship between CM and relationship satisfaction, IPV, and psychological distress. For gender, effect sizes were grouped by women and men, and tests of homogeneity between genders were conducted to determine if the mean effect size differed across women and men. We used a mixed-effects model for the moderation analysis, a random-effects model within subgroups, and a fixed-effect model across subgroups (Borenstein et al., 2010). For the three continuous moderators (i.e., mean age, proportion of cultural diversity, and publication year) univariate meta-regression models were used.

## Results

### Study Selection

**Figure 1.**
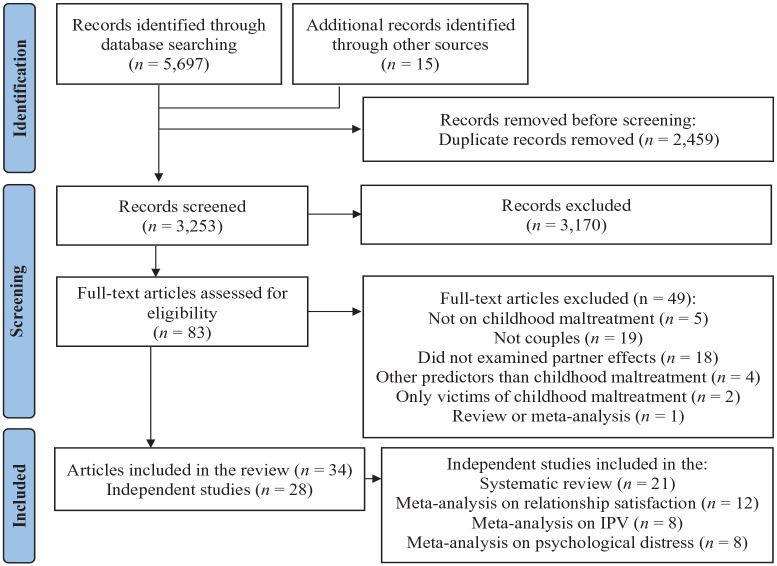
Preferred Reporting Items for Systematic Reviews and Meta-Analyses (PRISMA) flow diagram of study selection.

Each step of the process of study selection is presented in Figure 1 (Page et al., 2021). Overall, 34 articles met eligibility criteria, including 28 independent samples. Based on the outcomes, 21 studies were included in the systematic review, 12 in the meta-analysis on relationship satisfaction, 8 in the meta-analysis on IPV, and 8 in the meta-analysis on psychological distress. As most studies included more than one outcome (see Table 1), they could be included in the review and the meta-analyses or in more than one meta-analysis.

**Table 1.** Summary of the Reviewed Studies.

#. Authors, Year	Sample (*M*_age_) (% CD)	Types of CM	Outcomes	*r*	Main Results
1. Arbel et al. (2016)	91 heterosexual married couples (46.90) (71%)	- Family of origin aggression: CEA, CPA, interparental verbal and physical aggression	- Hostility during family conflict discussion - *Depressive symptoms*^c^ - Cortisol reactivity	.12	Women and men’s family-of-origin aggression were not significantly related to their partner’s observed hostility during in-lab family conflict discussion and to their partner’s depressive symptoms. Women’s family-of-origin aggression was associated with their male partner’s higher cortisol reactivity, whereas men’s family-of-origin aggression was not significantly related to their female partner’s cortisol reactivity.
2. Bai and Han (2016)	194 heterosexual couples with children (38.94) (100%)	- CEA	- Emotion dysregulation - *Parenting stress*^c^	.22	Women and men’s CEA were related to their partner’s higher emotion dysregulation. Men’s CEA, but not women’s, was related to their partner’s higher parenting stress.
3. Banford Witting and Busby (2022)	3,836 heterosexual couples in an exclusive relationship for at least 1 year (30.11) (20%)	- CPA - CSA	- Attachment behaviors - Coming to terms with the family of origin history	−.15	Women and men’s CPA and CSA were related to their partner’s lower attachment behaviors and their partner’s lower feeling of coming to terms with what happened in their family of origin.
3. Busby et al. (2011)	5,400 heterosexual couples (29.40) (13%)	- CPA	- Negative communication - Neuroticism		Women and men partnered with a person reporting CPA had similar levels of negative communication and neuroticism than those partnered with a person without a CPA history.
3. Knapp et al. (2017)	2,314 heterosexual couples (28.73) (14%)	- CSA	- *Relationship satisfaction*^a^ - Relationship instability		Women’s incestuous CSA was related to their male partner’s lower relationship satisfaction but was not significantly related to their male partner’s relationship instability.
3. Walker et al. (2011)	10,061 heterosexual couples (29.80) (13%)	- CSA	- Contempt and defensiveness		Women and men partnered with an individual reporting CSA had higher levels of contempt and defensiveness than couples where both partners had no CSA history.
4. Celsi et al. (2021)	134 non-cohabiting heterosexual couples (22.69) (4%)	- CPA - CEA - CPN - CEN	- *Perpetrated or suffered cyber dating abuse*^b^: pressure-aggression and monitoring-control subscales	.10	All forms of women and men’s childhood abuse and neglect were not significantly related to their partner’s higher perpetration of pressure-aggression cyber dating abuse. Men and women’s CEA were related to their partner’s higher perpetration of monitoring-control cyber dating abuse, whereas other forms of women and men’s childhood abuse and neglect were not significantly related. Only men’s CEA was related to their female partner’s higher victimization of pressure-aggression cyber dating abuse, whereas other forms of childhood abuse and neglect as well as women’s CEA were not significantly related. Men’s CPN and women’s CEA were related to their partner’s higher victimization of monitoring-control cyber dating abuse, whereas other forms of men and women’s childhood abuse and neglect were not significantly related.
5. Corsini-Munt et al. (2017)	49 cohabiting crossed-sex couples (28.92) (NR)	- CM: CEA, CPA, CSA, CEN, CPN	- *Anxiety*^c^ **-** *Relationship satisfaction*^a^ - Pain during sexual intercourse - Sexual function	.21 −.19	Women’s CM was related to their male partner’s lower sexual function, whereas men’s CM was not significantly related to their female partner’s sexual function. Men and women’s CM were not significantly related to their partner’s relationship satisfaction. Men’s CM was related to their female partner’s higher anxiety whereas women’s CM was not significantly related to their male partner’s anxiety. Men’s CM was related to their female partner’s higher affective pain during sexual intercourse, but it was not significantly related to sensory pain.
6. DiLillo et al. (2016)	200 heterosexual newlywed couples (26.56) (7%)	- CSA	*- Trauma symptoms*^c^ *- Dysphoria (irritability, depression, anxiety)*^c^ - Self-dysfunction	.13	Women and men’s CSA were related to their partner’s higher trauma symptoms but were not significantly related to their partner’s trauma dysphoria (general distress or dysphoric moods). Men’s CSA was related to their female partner’s trauma self-dysfunction symptoms (sexual-related problems and conflicts and maladaptive attempts to cope with negative affect) whereas women’s CSA was not significantly related to their male partner’s trauma self-dysfunction symptoms.
6. Evans et al. (2014)	193 heterosexual newlywed couples (26.59) (6%)	- CPA	- *Trauma symptoms*^c^		Women and men’s CPA were not significantly related to their partner’s trauma symptoms.
7. Dugal et al. (2020)	501 heterosexual couples (50.00) (NR)	- CM: CEA, CPA, CSA, CEN, CPN, witnessing interparental violence, bullying	- Negative urgency - *Perpetrated psychological IPV*^b^	.17	Women and men’s CM were related to their partner’s higher negative urgency and their partner’s higher perpetration of psychological IPV.
8. Fritz et al. (2012)	453 married or cohabiting heterosexual couples with at least one child (36.00) (19%)	- CPA	- *Physical IPV victimization and perpetration*^b^	.01	Higher reports of father-to-child CPA was related to higher levels of partner’s reports of IPV victimization and perpetration. Higher reports of mother-to-child CPA was related to lower levels of partner’s reports of IPV perpetration and was not significantly related to partner’s reports of IPV victimization.
9. Georgia et al. (2018)	701 heterosexual couples (35.48) (40%)	- CSA	- *Relationship satisfaction*^a^	−.00	Women’s CSA was not significantly related to their male partner’s relationship satisfaction.
10. Godbout et al. (2009)	304 heterosexual couples (28.55) (NR)	- CPA - CEA	- Attachment anxiety and avoidance - *Psychological and physical IPV perpetration*^b^ - *Relationship satisfaction*^a^	.09 −.06	Women’s CPA and CEA were related to their male partner’s higher attachment anxiety and their male partner’s higher perpetration of psychological IPV. Men’s CEA, but not men’s CPA, was related to their female partner’s higher perpetration of psychological IPV. Women and men’s CPA and CEA were not significantly related to their partner’s attachment avoidance, perpetration of physical IPV, and relationship satisfaction and men’s CPA and CEA were not significantly related to their partner’s attachment anxiety.
11. Godbout et al. (2023)	843 different-gender couples parent of a child younger than 12 months (31.60) (NR)	- Childhood interpersonal trauma: CEA, CPA, CSA, CEN, CPN, exposure to interparental violence, bullying	*- Postpartum depressive symptoms*^c^ - Mindfulness	.10	Mothers and fathers’ childhood interpersonal trauma were not significantly related to their partner’s mindfulness, but was significantly related to their partner’s higher postpartum depressive symptoms.
12. Johnson et al. (2020)	400 heterosexual couples (28.50) (35%)	- Adverse child experiences: CEA, CPA	- *Psychological IPV perpetration*^b^	.06	A person’s adverse childhood experiences was related to their partner’s higher levels of perpetration of psychological aggression.
13. Kazmierski et al. (2020)	112 opposite-sex couples (22.60) (73%)	- Family-of-origin aggression: CEA, CPA	- Cortisol levels (hypothalamic-pituitary-adrenal reactivity)		Women and men’s family-of-origin aggression were not significantly related to their partner’s hypothalamic-pituitary-adrenal reactivity (intercepts and the slopes of salivary cortisol levels) during emotionally vulnerable interactions between partners.
14. Liu et al. (2019)	156 first-time parent couples during the transition to parenthood (32.09) (NR)	- Child emotional maltreatment: CEA, CEN	- *Relationship satisfaction*^a^ - Emotion regulation strategies: cognitive reappraisal and expressive suppression - *Depression*^c^ - *Anxiety*^c^ - *Stress*^c^	−.08 −.02	Men’s childhood emotional maltreatment was related to their female partner’s lower reports of cognitive reappraisal whereas women’s childhood emotional maltreatment was not significantly related to their male partner’s cognitive reappraisal. Women and men’s childhood emotional maltreatment were not significantly related to their partner’s relationship satisfaction, expressive suppression, depressive symptoms, anxiety symptoms, and level of stress.
15. Mair et al. (2012)	1,861 married or cohabiting heterosexual couples (41.80) (NR)	- Adverse child experience: CEA, CPA, CSA, exposure to a mentally ill or alcoholic parent, violence against the mother	- *Physical IPV perpetration*^b^ - Impulsivity - Frequency of intoxication - *Depression*^c^ - *Anxiety*^c^	.06 .04	Women’s adverse childhood experience was related to their male partner’s higher perpetration of physical IPV whereas it was not significantly related to their male partner’s impulsivity, frequency of intoxication, depressive symptoms, and anxiety symptoms. Men’s adverse childhood experience was related to their female partner’s higher impulsivity, higher frequency of intoxication, and higher anxiety symptoms whereas it was not significantly related to their female partner’s perpetration of physical IPV and depressive symptoms.
16. Maleck and Papp (2015)	100 heterosexual dating couples (20.65) (17%)	- Risky family environment: CEA, CPA, CEN, CPN, exposure to interparental violence	- Observed positive and negative interactions - *Relationship satisfaction*^a^	.12	Men’s childhood risky family environment was related to their female partner’s lower observed positive interactions (e.g., support, engagement, communication skills) and higher observed negative interactions (e.g., conflict behavior, negative affect) during an in-lab conflictual discussion whereas women’s childhood risky family environment was not significantly related to their male partner’s positive and negative conflict interactions. Women and men’s childhood risky family environments were not significantly related to their partner’s relationship satisfaction.
17. Maneta et al. (2012)	109 heterosexual cohabiting couples (32.45) (42%)	- CPA - CSA - CEA	- *Physical IPV perpetration*^b^	.13	Women and men’s CPA were related to their partner’s higher perpetration of physical IPV. Women and men’s CSA and CEA were not significantly related to their partner’s perpetration of physical IPV.
17. Maneta et al. (2015)	156 heterosexual cohabiting couples (37.25) (29%)	- CEA	- *Relationship satisfaction*^a^ - Empathic accuracy for hostile emotions	−.18	Women and men’s CEA were related to their partner’s lower relationship satisfaction. Women’s CEA was related to their male partner’s lower empathic accuracy for hostile emotions whereas men’s CEA was not significantly related to their female partner’s empathic accuracy for hostile emotions.
18. Millwood (2011)	59 heterosexual couples (31.70) (16%)	- CSA	-Empathic accuracy for partner’s feelings -Emotional numbing		Male partners of women with a history of CSA reported higher emotional numbing and were more likely to be in the high empathic accuracy group during an in-lab conflictual discussion compared with male partners of women without a history of CSA.
19. Nelson and Wampler (2000)	161 heterosexual couples (32.41) (19%)	- CPA and/or CSA	- *Relationship satisfaction*^a^ - *Stress symptoms*^c^ - Family adjustment: cohesion and adaptability	−.12 .27	Men partnered with a woman reporting CPA and/or CSA had lower relationship satisfaction than men in the no-abuse group, whereas women partnered with a man reporting CPA and/or CSA had similar levels of relationship satisfaction than women in the no-abuse group. Women and men partnered with a person reporting CPA and/or CSA had higher individual stress symptoms than men and women in the no-abuse group. Women and men partnered with a person reporting CPA and/or CSA had similar levels of family cohesion and adaptability than women and men in the no-abuse group.
20. Nguyen et al. (2017)	414 heterosexual newlywed couples (27.10) (88%)	- Child abuse: CPA, CSA	- *Relationship satisfaction*^a^	−.03	Women and men’s child abuse were not significantly related to their partner’s relationship satisfaction.
21. Peterson et al. (2018)	52 heterosexual couples (20.08) (29%)	- Child emotional maltreatment: CEA, CEN	- *Relationship satisfaction*^a^	−.06	Men’s childhood emotional maltreatment was related to their female partner’s lower relationship satisfaction, whereas women’s childhood emotional maltreatment was not significantly related to their male partner’s relationship satisfaction.
22. Riggs et al. (2011)	155 heterosexual dating couples (21.91) (28%)	- CEA	- *Relationship satisfaction*^a^ - Attachment avoidance and anxiety	−.13	A person’s CEA was related to their partner’s lower relationship satisfaction but was not significantly related to their partner’s attachment avoidance and anxiety.
23. Steel et al. (2017)	53 heterosexual dating couples (19.47) (17%)	- CPA	- *Physical IPV perpetration*^b^	.19	Women’s CPA was related to their male partner’s higher physical IPV perpetration whereas men’s CPA was not significantly related to their female partner’s physical IPV perpetration.
24. Vaillancourt-Morel et al. (2019)	365 mixed-sex couples (28.59) (NR)	- CM: CEA, CPA, CSA, CEN, CPN	- Self-disclosure, partner disclosure, perception of partner empathic responses - Sexual satisfaction - *Relationship satisfaction*^a^	−.18	Women’s CM was related to their male partner’s lower perception of partner’s empathic responses, lower sexual satisfaction, and lower relationship satisfaction, but it was not significantly related to their male partner’s self-disclosure and perception of partner disclosure. Men’s CM was related to their female partner’s lower relationship satisfaction, but it was not significantly related to their partner’s self-disclosure, perception of partner disclosure and empathic responses, and sexual satisfaction.
25. Vaillancourt-Morel et al. (2021)	269 mixed-sex couples (28.76) (NR)	- CEA - CPA - CSA - CEN - CPN	- Sexual satisfaction - Sexual function - Sexual distress		Men’s CEA and CEN were significantly related to their female partner’s higher sexual distress, but they were not significantly related to their partner’s sexual satisfaction and sexual function. Women’s CEA was significantly related to their male partner’s lower sexual function, but it was not significantly related to their partner’s sexual satisfaction and sexual distress. Men and women’s CPA, CSA, and CPN were not significantly related to their partner’s sexual satisfaction, sexual function, and sexual distress. Men and women’s CPA, CSA, CEA, CPN, CEN were not significantly related to their partner’s trajectories of sexual satisfaction, sexual function, and sexual distress over 1 year.
26. Vaillancourt-Morel et al. (2023)	228 mixed- and same-sex couples (30.44) (9.6%)	- CM: CEA, CPA, CSA, CEN, CPN	- Perceived partner responsiveness		A person’s cumulative CM was significantly related to their partner’s greater day-to-day variability in their perception of partner responsiveness, but was not significantly related to their partner’s mean perception of partner responsiveness over 35 days, partner responsiveness at Time 1, and partner responsiveness over 1 year.
27. Whisman (2014)	2,161 married heterosexual couples (66.20) (11%)	- CPA	- Perceived positive and negative marital exchanges		A person’s CPA was related to their partner’s lower perception of positive marital exchanges but was not significantly related to their partner’s perception of negative exchanges.
28. Winer et al. (2018)	218 mixed-sex newlywed couples (28.42) (6%)	- Childhood family adversity: CEA, CPA CEN, CPN, family conflict, household dysfunction	- Negative and positive behaviors - Cortisol levels (hypothalamic-pituitary-adrenal reactivity)		Women’s childhood family adversity was associated with their male partner’s attenuated cortisol response to in-lab conflict discussion whereas men’s childhood family adversity was not significantly associated with their female partner’s cortisol response. Women and men’s childhood family adversity were not significantly related to their partner’s negative and positive behaviors during an in-lab conflict discussion.
29. Zhang et al. (2022)	163 mixed-sex couples of Black Americans (29.90) (100% for one partner, NR for the other partner)	Childhood adversity: CPA, CPN, CSA, household dysfunction, bullying, racial discrimination	- Chronic illness - Aging		Women and men’s adverse childhood experiences were significantly related to their partner’s higher chronic illness and higher accelerated aging.

*Note.* Outcomes in italic are those included in the meta-analysis. % CD = proportion of cultural diversity among the total sample; NR = not reported; CM = childhood maltreatment; CSA = child sexual abuse; CPA = child physical abuse; CEA = child emotional/psychological abuse; CEN = child emotional neglect; CPN = child physical neglect; IPV = intimate partner violence.

a Outcome included in the effect size for relationship satisfaction.

b Outcome included in the effect size for IPV.

c Outcome included in the effect size for psychological distress.

### Study Quality and Characteristics

Table 1 reports the characteristics of the 34 included studies (28 independent samples), that is, sample size and characteristics, types of CM and partner’s outcomes examined, effect size if included in the meta-analysis, and a description of main finding. As all studies included were dyadic and peer-reviewed, they were all similar in the methodology used (i.e., correlational, non-representative sample, validated tools) and reached minimal quality standards. Methodological characteristics that varied between studies (i.e., sample’s mean age, proportion of cultural diversity, and publication year) were examined as proxy of study quality and used as moderators in the meta-analyses. Studies were published between 2000 and 2023. Most studies were conducted in the United States of America (*k* = 20), six in Canada, and two in China. Sample size ranged from 49 to 10,061 couples (median *N* = 197), with a total of 40,690 participants (20,345 couples). Participants’ mean age was 31.96 years (*SD* = 10.08; range = 19.47–66.20). Most studies used a convenience sample of couples (*k* = 24) and four used a representative sample. Most couples were recruited in the community (*k* = 25) whereas three studies recruited clinical samples (i.e., couples who sought couple therapy, couples including a woman with pain during sexual intercourse, couples seeking to enroll in an online self-help program). As all studies only recruited mixed-sex couples (mostly described as heterosexual couples), except one in which 52.4% of participants were women, 50.1% of the participants were women, and 49.9% were men. Relationship status of participants varied widely with seven studies lumping all relationship status together (e.g., married, cohabiting, and non-cohabiting couples), seven studies including specifically dating or non-cohabiting couples, eight studies including only cohabiting couples regardless of their marital status, and six studies including only married couples (three only newlyweds). Couples’ average relationship length, based on the 17 studies reporting this information, was 5.63 years (*SD* = 4.86; range = 1.51, 22). Mean proportion of cultural diversity in the sample, based on the 20 studies reporting this information, was 31.53% (*SD* = 28.66; range = 3.50–100.00). All studies (*k* = 28) used a retrospective design with young adults or adults and almost all studies were cross-sectional (*k* = 26) except two longitudinal studies with a 1-year follow-up.

### Systematic Review on the Associations Between a Person’s CM and Their Partner’s Couple Outcomes

#### Couple Functioning

Two studies examined how a person’s CM was related to their partner’s overall couple functioning outside of relationship satisfaction, specifically relationship instability and family cohesion and adaptability. These associations were all non-significant as women’s incestuous CSA was not significantly related to their male partner’s relationship instability (Knapp et al., 2017) and women and men’s CPA and/or CSA were not significantly related to their partner’s levels of family cohesion and adaptability (Nelson & Wampler, 2000).

#### Communicative Behaviors and Perceptions

Nine studies examined how a person’s CM was related to their partner’s communicative behaviors including emotions and how a person perceived others’ behaviors, thoughts, and feelings. The outcomes examined included negative and positive communication or exchanges, observed hostility, contempt and defensiveness, empathic accuracy (i.e., how accurately one person can infer the thoughts and feelings of their partner), self-disclosure, and perceived empathic responses (i.e., partner responsiveness). Seven studies found significant associations. Men’s adverse childhood experiences were related to their female partners’ lower observed positive interactions (e.g., support, engagement, communication skills) and higher observed negative interactions (e.g., conflict behavior, negative affect) during an in-lab conflictual discussion (Maleck & Papp, 2015). A person’s CPA was related to their partner’s lower self-reported perception of positive marital exchanges (Whisman, 2014), women’s CM was related to their partner’s lower self-reported perception of partner’s empathic responses (Vaillancourt-Morel et al., 2019), a person’s CM was related to their partner’s greater day-to-day variability in their perception of partner responsiveness (Vaillancourt-Morel et al., 2023), and women and men’s CSA was related to their partner’s self-reported higher levels of contempt and defensiveness (Walker et al., 2011). Two studies found opposite significant associations with how a person perceived emotions in others during couple interactions. Women’s CEA was related to their partner’s lower empathic accuracy for hostile emotions during an in-lab conflictual discussion (Maneta et al., 2015), whereas women’s CSA was related to their partner’s higher empathic accuracy for overall feeling state during an in-lab conflictual discussion (Millwood, 2011).

However, eight studies reported non-significant associations. Women and men’s childhood family adversity were not significantly related to their partner’s negative and positive behaviors during an in-lab conflict discussion (Winer et al., 2018) and women and men’s family-of-origin aggression were not significantly related to their partner’s observed hostility during an in-lab family conflict discussion (Arbel et al., 2016). Moreover, a person’s CPA was not significantly related to their partner’s self-reported perception of negative marital exchanges (Whisman, 2014), to their partner’s self-reported levels of negative communication (Busby et al., 2011), women and men’s CM was not significantly related to their partner’s self-disclosure and perception of partner disclosure (Vaillancourt-Morel et al., 2019), and a person’s CM was not significantly related to their partner’s mean perception of partner responsiveness over 35 days, level of partner responsiveness at Time 1, and the trajectory of partner responsiveness over 1 year (Vaillancourt-Morel et al., 2023). Similarly, but for women only, women’s adverse childhood experience was not significantly related to their partners’ observed positive and negative interactions during an in-lab conflictual discussion (Maleck & Papp, 2015). Finally, men’s CEA was not significantly related to their partner’s empathic accuracy for hostile emotions (Maneta et al., 2015) and men’s CM was not significantly related to their partner’s lower self-reported perception of partner’s empathic responses (Vaillancourt-Morel et al., 2019).

#### Attachment

Three studies examined how a person’s CM was related to their partner’s romantic attachment behaviors and showed mixed findings. Women and men’s CPA and CSA were significantly related to their partner’s lower attachment behaviors (accessibility, responsiveness, and engagement) (Banford Witting & Busby, 2022) and women’s CPA and CEA were related to their partner’s higher attachment anxiety (Godbout et al., 2009). However, women’s CPA and CEA were not significantly related to their partner’s higher attachment avoidance, men’s CPA and CEA were not significantly related to their partner’s attachment anxiety and avoidance (Godbout et al., 2009), and a person’s CEA was not significantly related to their partner’s attachment avoidance and anxiety (Riggs et al., 2011).

#### Sexuality

Two studies examined how a person’s CM was related to their partner’s sexuality including sexual function, sexual distress, sexual satisfaction, and pain during sexual intercourse and showed mixed findings. Among a sample of women reporting pain during sexual intercourse and their partners, women’s CM was related to their partner’s lower sexual function, whereas men’s CM was not significantly related to partner’s sexual function (Corsini-Munt et al., 2017). Men’s CM was related to their partner’s higher affective pain during sexual intercourse, but it was not significantly related to sensory pain (Corsini-Munt et al., 2017). Among a sample of community couples followed over 1 year, women’s CM was related to their partner’s lower sexual satisfaction, but men’s CM was not significantly related to their partner’s sexual satisfaction (Vaillancourt-Morel et al., 2019). Taking each type of CM separately, men’s CEA and CEN were significantly related to their partner’s higher sexual distress, but they were not significantly related to their partner’s sexual satisfaction and sexual function (Vaillancourt-Morel et al., 2021). Women’s CEA was significantly related to their partner’s lower sexual function, but it was not significantly related to their partner’s sexual satisfaction and sexual distress (Vaillancourt-Morel et al., 2021). Men and women’s CPA, CSA, and CPN were not significantly related to their partner’s sexual satisfaction, sexual function, and sexual distress (Vaillancourt-Morel et al., 2021). Men and women’s CPA, CSA, CEA, CPN, CEN were not significantly related to their partner’s trajectories of sexual satisfaction, sexual function, and sexual distress over 1 year (Vaillancourt-Morel et al., 2021).

### Systematic Review on the Associations Between a Person’s CM and Their Partner’s Individual Outcomes

#### Emotion Reactivity and Regulation Strategies

Seven studies examined how a person’s CM was related to their partner’s emotion reactivity and regulation strategies. The outcomes examined included emotional numbing (i.e., difficulty feeling emotions), neuroticism (i.e., trait disposition to experience negative affects), negative urgency (i.e., inability to refrain from using rash and maladaptive behaviors when experiencing negative affect despite their possible negative consequences), mindfulness (i.e., paying attention on purpose and non-judgmentally to the unfolding of emotions and experience), and diverse emotion regulation strategies (e.g., cognitive reappraisal, expressive suppression, impulsive behaviors, intoxication, coming to terms). Six studies reported significant associations. Women and men’s CM were related to their partner’s negative urgency (Dugal et al., 2020) and women and men’s CPA and CSA were significantly related to their partner’s lower feeling of coming to terms with what happened in their family of origin (Banford Witting & Busby, 2022). Women’s CSA was related to their partner’s higher emotional numbing during an in-lab conflictual discussion (Millwood, 2011). Men’s childhood emotional maltreatment (CEA and CEN) was related to their partner’s lower reports of cognitive reappraisal, an emotion regulation strategy (Liu et al., 2019), men’s CSA was related to their partner’s maladaptive attempts to cope with negative affect (DiLillo et al., 2016), and men’s adverse childhood experience was related to their partner’s higher impulsivity and frequency of intoxication (Mair et al., 2012).

Five studies reported non-significant associations. Women and men’s childhood emotional maltreatment (CEA and CEN) were not significantly related to their partner’s expressive suppression, an emotion regulation strategy (Liu et al., 2019), women and men’s CPA were not significantly related to their partner’s levels of neuroticism (Busby et al., 2011), and women and men’s childhood interpersonal trauma were not significantly related to their partner’s mindfulness (Godbout et al., 2023). Women’s adverse childhood experiences were not significantly related to their partner’s impulsivity and frequency of intoxication (Mair et al., 2012), women’s childhood emotional maltreatment was not significantly related to their partner’s reports of cognitive reappraisal, an emotion regulation strategy (Liu et al., 2019), and women’s CSA was not related to their partner’s maladaptive attempts to cope with negative affect (DiLillo et al., 2016).

#### Stress Reactivity and Health Outcomes

Four studies examined how a person’s CM was related to their partner’s health outcomes including stress reactivity, chronic illness, and aging. Three studies examined how a person’s CM was related to their partner’s hypothalamic-pituitary-adrenal reactivity using cortisol levels during in-lab family or couple’s interactions with nonsignificant results for men’s CM and mixed findings for the effect of women’s CM. Indeed, women’s family-of-origin aggression was significantly associated with their partner’s higher cortisol reactivity during in-lab family conflict discussion (Arbel et al., 2016), women’s childhood family adversity was associated with their partner’s attenuated cortisol response during an in-lab conflict discussion (Winer et al., 2018), and women’s family-of-origin aggression was not significantly related to their partner’s hypothalamic-pituitary-adrenal reactivity (intercepts and the slopes of salivary cortisol levels) during in-lab emotionally vulnerable interactions between partners (Kazmierski et al., 2020). Men’s family-of-origin aggression and men’s childhood family adversity were not significantly related to their partner’s cortisol response during in-lab interactions (Arbel et al., 2016; Kazmierski et al., 2020; Winer et al., 2018). Another study showed that a person’s adverse childhood experiences was significantly related to their partner’s higher chronic illness and accelerated aging (Zhang et al., 2022).

### Meta-Analysis on the Associations Between a Person’s CM and Their Partner’s Outcomes

#### Relationship Satisfaction

Twelve studies examined how a person’s CM was related to their partner’s relationship satisfaction. Results for the effect sizes and the moderation analyses are presented in Table 2. Results of the meta-analysis using the 12 studies showed that a person’s CM is significantly related to their partner’s lower relationship satisfaction, *k* = 12; *N* = 25 348; *r* = −.09, 95% CI = [−0.14, −0.04], *p* = .001, but this association did not reach the magnitude of a small effect. This association was significant and similar for women and men’s CM as gender did not moderate the effect size. Moreover, no significant difference was found for the size of the effect as a function of sample’s mean age, proportion of cultural diversity, and publication year.

**Table 2.** Effect Sizes of the Associations Between a Person’s Childhood Maltreatment and their Partner’s Relationship Satisfaction, Intimate Partner Violence, and Psychological Distress.

Moderators	*k*	*N*	*r*	[95% CI]	*z* (*p)*	*Q* (*p*)	*I* ^2^	*Q*′ (*p*)	Slope (*p*)
Relationship satisfaction									
All studies	12	25,348	−.09	[−0.14, −0.04]	−3.42 (.001)	33.72 (<.001)	67.38		
Sex									
Women	11	12,519	−.07	[−0.13, −0.01]	−2.33 (.020)				
Men	9	1,757	−.10	[−0.17, −0.04]	−3.16 (.002)				
Contrast analysis								0.52 (.469)	
Age of the sample	12	25,348							−0.001 (.735)
Proportion of cultural diversity	8	5,490							0.088 (.544)
Year of publication	12	25,348							−0.001 (.868)
Intimate partner violence									
All studies	8	7,724	.08	[0.05, 0.12]	4.59 (<.001)	14.84 (.038)	52.82		
Sex									
Women	6	3,009	.10	[0.06, 0.13]	5.17 (< .001)				
Men	6	3,009	.09	[0.03, 0.15]	3.10 (.002)				
Contrast analysis								0.002 (.963)	
Age of the sample	8	7,724							0.001 (.583)
Proportion of cultural diversity	5	2,392							−0.027 (.899)
Year of publication	8	7,724							0.004 (.361)
Psychological distress									
All studies	8	7,110	.11	[0.06, 0.16]	4.03 (<.001)	24.58 (.001)	71.52		
Sex									
Women	8	3,555	.08	[0.03, 0.13]	3.08 (.002)				
Men	8	3,555	.12	[0.06, 0.18]	3.70 (<.001)				
Contrast analysis								0.83 (.361)	
Age of the sample	8	7,110							−0.003 (.517)
Proportion of cultural diversity	4	1,292							−0.035 (.730)
Year of publication	8	7,110							−0.007 (.194)

*Note.* CI = confidence interval.

#### Intimate Partner Violence

Eight studies examined how a person’s CM was related to their partner’s IPV including psychological and physical IPV perpetration, physical IPV victimization, and perpetrated and sustained cyber dating abuse. Only two studies examined IPV victimization whereas all studies examined perpetration, thus it was impossible to examine these forms of IPV separately and they were combined in the meta-analysis. Results for the effect sizes and the moderation analyses are presented in Table 2. Results of the meta-analysis using the eight studies as the unit of analysis showed that a person’s CM is significantly related to their partner’s higher IPV, *k* = 8; *N* = 7,724; *r* = .08, 95% CI = [0.05, 0.12], *p* = .001, but this association did not reach the magnitude of a small effect. This association was significant and similar for women and men’s CM as gender did not significantly moderate the effect size. Moreover, no significant difference was found for the size of the effect as a function of sample’s mean age, proportion of cultural diversity, and publication year.

#### Psychological Distress

Eight studies examined how a person’s CM was related to their partner’s psychological distress including general distress, trauma, depressive and anxious symptoms, level of stress, and emotion dysregulation. Results for the effect sizes and the moderation analyses are presented in Table 2. Results of the meta-analysis using the eight studies as the unit of analysis showed that a person’s CM is significantly related to their partner’s higher psychological distress, *k* = 8; *N* = 7,110; *r* = .11, 95% CI = [0.06, 0.16], *p* < .001, with a small effect size. This association was significant and similar for women and men’s CM as gender did not moderate the effect size. Moreover, no significant difference was found for the size of the effect as a function of sample’s mean age, proportion of cultural diversity, and publication year.

#### Publication Biases

Publication bias occurs when studies with statistically significant results are more likely to be published than studies with non-significant or unfavorable results. To estimate the likelihood of this effect for the three meta-analyses, we first examined the funnel plots of included studies (Sterne & Egger, 2001). The funnel plots were distributed symmetrically, suggesting an absence of publication bias. Then, we conducted the trim-and-fill test with a random effect which identifies and corrects for funnel plot asymmetry by trimming the studies that cause an asymmetry or filling the missing studies to offer a bias-corrected overall effect estimate (Duval & Tweedie, 2000). Results suggested that one study was missing in the funnel plot for relationship satisfaction and one for IPV, but the bias-corrected effect sizes remained similar. The fail-safe *N* suggested that a total of 117 (relationship satisfaction), 94 (IPV), and 64 (psychological distress) null studies would be necessary to reduce the effect sizes to non-significant values. These findings indicated little evidence for the presence of publication bias.

## Discussion

This systematic review and meta-analysis synthesized the research on partner effects of CM and identified 28 independent samples (34 articles) that examined the associations between a person’s CM and their partner’s outcomes. Overall, the systematic review shed light on the diversity of partner’s couple and individual outcomes that may be affected by a person’s CM, but more importantly, it shows that findings are mixed with some studies reporting negative partner effects and others, non-significant associations. Meta-analytic results showed significant, but trivial to small associations between a person’s CM and their partner’s lower relationship satisfaction (*r* = −.09), higher intimate partner violence (*r* = .08), and higher psychological distress (*r* = .11). Even if some past studies reported that the effects of CM on victims were different between women and men (Gershon et al., 2008), the effects on their partners were similar for women and men. Moreover, the methodological characteristics that were most often reported and varied between the included studies did not seem to contribute to the ability to detect significant partner effects as the meta-analytic associations did not differ as a function of sample’s mean age, proportion of cultural diversity, and publication year.

### Associations Between a Person’s CM and Their Partner’s Couple Outcomes

This systematic review offers an overall overview of the diversity of partner’s couple outcomes that have been examined in past studies as potentially affected by a person’s CM, that is, relationship instability and satisfaction, family cohesion and adaptability, negative and positive communication or exchanges, observed hostility, contempt and defensiveness, IPV, empathic accuracy, self-disclosure, perceived empathic responses, attachment, sexual function, sexual distress, sexual satisfaction, and pain during sexual intercourse. The systematic review suggests no meaningful associations between a person’s CM and their partner’s overall measures of couple functioning as there was no significant associations in the two studies examining the partner effects on general measures of couple functioning (i.e., relationship instability, family cohesion, and adaptability). However, the meta-analysis combining twelve studies shows a significant but weak (i.e., not reaching an effect of a small magnitude) association between a person’s CM and their partner’s relationship satisfaction (*r* = −.09, 95%CI [−0.14, −0.04]). A recent meta-analysis reported that emotional maltreatment is negatively related to victim’s later romantic relationship well-being with an effect of a small magnitude (*r* = .14; Cao et al., 2020). As the size of the effect on the victim is small, the weak effect size for partner effect is not surprising. Indeed, meta-analyses and a machine learning study have consistently shown that associations between a person’s experience and their own outcomes are stronger than the ones between a person’s experience and their partner’s outcomes (Candel & Turliuc, 2019; Joel et al., 2020). These overall measures of couple functioning including relationship satisfaction are general subjective assessments which may be more affected by intra-individual factors or more recent partner’s behaviors. The only previous meta-analysis that included the effect size for the partner effects showed that among four studies, childhood emotional maltreatment (CEA and CEN) was significantly related to partners’ romantic relationship well-being with a small effect size (*r* = −.13, 95% CI [−0.21, −0.05]; Cao et al., 2020). In comparison, our weak effect size seems a little lower, but still in the range of their confidence interval. However, for this effect size, Cao et al. (2020) combined only four studies, and merged different dimensions of relationship well-being (i.e., overall satisfaction, overall dysfunction, intimacy, conflicts, instability) which may explain their very large confidence interval. Thus, our meta-analysis combining 12 studies only for relationship satisfaction may be more accurate by suggesting a weak association.

Although overall couple functioning seems to be weakly related to one’s partner’s CM, seven studies reported that a person’s CM was significantly related to their partner’s communicative behaviors and perception including lower observed positive interactions, lower self-reported perception of positive marital exchanges and of partner’s empathic responses, and higher levels of contempt and defensiveness. However, in eight studies, a person’s CM was not significantly related to their partner’s communicative behaviors and perception. Thus, findings are still mixed with some of the significant associations being significant only in men or only in women and others only for specific forms of CM. These studies varied widely in their design (e.g., in-lab observational couple interactions, self-reported responses or perceptions), in the specific types of partner’s outcomes examined, and in the forms of CM included, which may explain for the mixed results between studies. Despite these mixed findings, some of these studies support that a person’s CM may not only affect their own behaviors, responses, and perceptions during couple discussions, but it may also be related to their partner’s, setting the stage for a reciprocal cycle of negative interactions. This supports the CATS theoretical model (Nelson & Smith, 2005; Nelson & Wampler, 2000) suggesting that a person’s CM may be related to dysfunctional dynamics within the couple system, including partners’ reports. Clinical case studies described how intimate relationship in adulthood CM may trigger cycles of repetition and enactment of traumatic interactions (MacIntosh, 2017). These potential bidirectional negative interactions could explain our meta-analytical result combining eight studies that show a significant but weak association between a person’s CM and their partner’s IPV (*r* = .08, 95% CI [0.05, 0.12]). The consequences of CM may create negative and hostile interactions in which both partners have negative behaviors, perceptions, and responses which may lead to problematic relational dynamics including perpetration of violence. Only two studies examined IPV victimization and all studies (*n* = 8) examined IPV perpetration. Thus, it was statistically impossible to determine if partners are more subject to perpetrate IPV or being the victim of it. As past meta-analyses have shown that a person’s CM is significantly related to their own higher perpetration and victimization of IPV with an effect of a small magnitude (*r* = .19; Godbout et al., 2019; *r* = .18; Li et al., 2019; *r* = .21; Smith-Marek et al., 2015), most partners might be both perpetrators and victims.

The associations between a person’s CM and their partner’s attachment and sexuality were examined in only three and two studies respectively, which underlines the need for more dyadic studies on these specific couple’s outcomes. In addition to the small number of studies, results were mixed as two studies showed that specific forms of CM were related to the partner’s attachment behaviors (i.e., higher attachment anxiety, lower accessibility, responsiveness, and engagement) whereas other studies found that the same form of CM and other forms were not significantly related to the partner’s attachment (i.e., attachment anxiety and avoidance). However, women’s CPA and CEA were not significantly related to their partner’s higher attachment avoidance, men’s CPA and CEA were not significantly related to their partner’s attachment anxiety and avoidance (Godbout et al., 2009), and a person’s CEA was not significantly related to their partner’s attachment avoidance and anxiety (Riggs et al., 2011). This mixed pattern of findings was similar for the partner’s sexual outcomes. These mixed results and the small number of studies limit the conclusion that can be drawn and underline the need to conduct more dyadic studies to better understand under which conditions a person’s CM may be negatively related to their partner’s attachment and sexuality.

### Associations Between a Person’s CM and Their Partner’s Individual Outcomes

This systematic review also offers an overall overview of the little diversity of partner’s individual outcomes that have been examined in past studies as potentially affected by a person’s CM, that is, general distress, trauma, depressive and anxious symptoms, level of stress, emotion dysregulation, emotional numbing, neuroticism, negative urgency, diverse emotion regulation strategies, chronic illness, and aging. Overall, our findings, combining results from eight studies, suggest an association of a small magnitude between a person’s CM and their partner’s higher psychological distress (*r* = .11, 95% CI [0.06, 0.16]). Moreover, six studies reported that a person’s CM was related to their partner’s higher emotion reactivity and maladaptive regulation strategies. These results show that it is not only the romantic functioning of victims and their partners that is affected by one partner’s CM, but that the partner may also report higher reactivity, difficulties regulating their emotions, and higher psychological distress that mimics victim’s trauma symptoms. This result is in line with the CATS Model (Nelson & Smith, 2005) that suggests that partners may report emotional and cognitive symptoms that are similar to the primary victim’s trauma response. This is also in line with the concept of secondary traumatic stress in which the demands of living and caring for someone who displays the symptoms of posttraumatic stress disorder would lead to the development of similar feelings and distress (Figley, 1998). Paired with our findings regarding dysfunctional relational dynamics and past studies on the effects of CM on victims (Lewis et al., 2021; Schar et al., 2022), these results outline that a person’s traumatic experiences may be related to psychological symptoms in the primary victim, but also secondary trauma psychological symptoms in the partner, and dysfunctional relational dynamics within the couple system. Thus, theoretically, the victim, their partner, and the couple dynamic may all be affected by a person’s CM and need to be taken into consideration in the development of appropriate preventive and intervention strategies.

However, five studies reported non-significant associations between a person’s CM, in particular women’s CM, and their partner emotion reactivity and regulation strategies. Moreover, the associations between a person’s CM and their partner’s stress reactivity were mixed, with non-significant effect for men’s CM and negative, positive, and non-significant associations for women’s CM. These mixed results on partner’s emotion reactivity, regulation strategies, and stress reactivity using cortisol levels suggest that moderating factors may be at play and explain for whom, for which form of CM, or in which specific context a person’s CM may be related to these outcomes in partners. Moreover, only one study recently examined the association between a person’s CM and their partner’s health reporting associations with their partner’s chronic illness and aging (Zhang et al., 2022). This is an important limitation that underlines the need to conduct more dyadic studies using mixed methods including biological assessment and a diversity of health outcomes.

### Limitations

The primary studies included in this review present some limitations. All studies included relied on retrospective self-reported data and almost all were cross-sectional. Thus, they could not provide information on causal associations between a person’s CM and their partner’s outcomes. The generalizability of our results is potentially limited as most studies included used a convenience sample of couples recruited in the community. Even if the proportion of cultural diversity was not significantly related to the effect sizes, most samples reported low ethnic diversity (mostly Caucasians) and all studies, except one, included only mixed-sex couples (men with women), which significantly limits generalization of the findings to other ethnic, gender, and sexual diversity groups. Moreover, even if most studies used statistical models that test simultaneously the effect of a person’s own CM and their partner’s CM, studies did not examine specifically if partner effects were more severe in dual-trauma couples, that is, couples wherein both partners have reported CM. The limitations of the meta-analysis comprise the rather small number of studies included (*k* = 8–12) which underlines the need for more dyadic studies examining partner effects of CM and lowers our confidence in the obtained effect sizes. Given this small number of studies available, it was impossible to compare the effect sizes across the different forms of CM or of IPV or across other potential moderators.

### Summary Table of Critical Findings

**Table:** 

• As only 28 dyadic studies were available, more studies using dyadic design are still needed. • The systematic review shows that findings are mixed with some studies reporting negative partner effects and others, non-significant associations for several outcomes. • Meta-analyses showed significant, but trivial to small associations between a person’s CM and their partner’s lower relationship satisfaction, higher IPV, and higher psychological distress. • Even if there is a diversity of partner’s individual and couple outcomes that have been examined in past studies, some outcomes were not examined thoroughly (e.g., sexuality, attachment, stress reactivity, health), and we need more studies using high-quality methods.

### Summary Table of Implication for Practice, Policy, and Research

**Table:** 

• Researchers should consider using a dyadic design to examine the association between a person’s CM and their partner’s outcomes in particular the outcomes that were understudied. • Researchers should consider using high-quality mixed methods that include longitudinal design, observational method, and biological assessment to get a full picture of how a person’s CM may be related to their partner’s outcomes. • Researchers should consider a wider range of ethnicities, sexual orientations, and genders. • Prevention and intervention strategies should acknowledge that a person’s CM may also affect their romantic partner and offer victims’ partners specific services. • In clinical practice, it may be valuable to develop trauma-focused couple approaches that take into consideration the effect of a person’s CM on the victim, the partner, and couple functioning.
